# TMS-Induced Controlled BBB Opening: Preclinical Characterization and Implications for Treatment of Brain Cancer

**DOI:** 10.3390/pharmaceutics12100946

**Published:** 2020-10-05

**Authors:** Udi Vazana, Lior Schori, Uri Monsonego, Evyatar Swissa, Gabriel S. Pell, Yiftach Roth, Pnina Brodt, Alon Friedman, Ofer Prager

**Affiliations:** 1 Departments of Physiology and Cell Biology, Cognitive and Brain Sciences, The Zlotowski Center for Neuroscience, Ben-Gurion University of the Negev, no.1 Ben-Gurion Blvd., Beer-Sheva 8410501, Israel; liorscho@post.bgu.ac.il (L.S.); urimons@post.bgu.ac.il (U.M.); swisse@post.bgu.ac.il (E.S.); alonf@bgu.ac.il (A.F.); pragero@post.bgu.ac.il (O.P.); 2 Department of Life Sciences, Zlotowski Center for Neuroscience, Ben-Gurion University of the Negev, no.1 Ben-Gurion Blvd., Beer-Sheva 8410501, Israel; pell@brainsway.com (G.S.P.); yiftah@brainsway.com (Y.R.); 3 Brainsway Ltd., 19th Hartom St., Jerusalem 9777518, Israel; 4 Department of Surgery, Oncology and Medicine, McGill University and the Research Institute-McGill University Health Center, 2155 Rue Guy, Montreal, QC H3H 2L9, Canada; pnina.brodt@mcgill.ca; 5 Department of Medical Neuroscience and the Brain Repair Centre, Faculty of Medicine, Dalhousie University, 5850 College St., Halifax, NS B3H 4R2, Canada

**Keywords:** blood–brain barrier, brain drug delivery, repetitive transcranial magnetic stimulation, safety, time dependency, IGF-Trap

## Abstract

Proper neuronal function requires strict maintenance of the brain’s extracellular environment. Therefore, passage of molecules between the circulation and brain neuropil is tightly regulated by the blood–brain barrier (BBB). While the BBB is vital for normal brain function, it also restricts the passage of drugs, potentially effective in treating brain diseases, into the brain. Despite previous attempts, there is still an unmet need to develop novel approaches that will allow safe opening of the BBB for drug delivery. We have recently shown in experimental rodents and in a pilot human trial that low-frequency, high-amplitude repetitive transcranial magnetic stimulation (rTMS) allows the delivery of peripherally injected fluorescent and Gd-based tracers into the brain. The goals of this study were to characterize the duration and safety level of rTMS-induced BBB opening and test its capacity to enhance the delivery of the antitumor growth agent, insulin-like growth factor trap, across the BBB. We employed direct vascular and magnetic resonance imaging, as well as electrocorticography recordings, to assess the impact of rTMS on brain vascular permeability and electrical activity, respectively. Our findings indicate that rTMS induces a transient and safe BBB opening with a potential to facilitate drug delivery into the brain.

## 1. Introduction

Proper neuronal function requires strict regulation over the molecular composition in the brain’s extracellular environment. Hence, the passage of molecules between the circulation and brain neuropil must be tightly regulated. The endothelium of brain blood vessels, through which molecular traffic takes place, together with neighboring pericytes and astrocytes, form a complex interface designed for this purpose, known as the blood–brain barrier (BBB) [1]. The BBB restricts the diffusion of ions, low-molecular weight hydrophilic molecules, toxins and pathogens across the vascular endothelium and forces required molecular traffic (to and from the brain) to take place in a transcellular manner, which is controlled through several transport mechanisms [1].

While an intact BBB is a key for normal brain function, its restrictive nature limits central nervous system (CNS) drug delivery and subsequent efficacy in treatment of brain diseases. In malignant brain tumors, for example, despite the often found increase in microvascular permeability within the tumor bed, the surrounding, intact BBB limits the transfer of most chemotherapeutics from blood to brain [1,2]. As a result, chemotherapeutics that are effective in limiting proliferation of tumor cells outside the CNS fail to do so when the tumor is within the brain [3,4]. Furthermore, the move toward targeted drug therapies in the form of immune-therapeutics and cytostatic agents is accompanied by the development of large molecules that do not reach the brain at effective concentrations. For example, insulin-like growth factor trap (IGF-Trap) was shown in preclinical trials to suppress the growth of liver metastases of colon and lung carcinoma [5,6], yet its high molecular weight (>400 KDa) restricts it from crossing the BBB for the treatment of brain metastases. It is therefore becoming clear that the success of ongoing efforts to develop pharmaceuticals targeted against CNS diseases will be limited if not accompanied by means to overcome the BBB’s restrictive nature.

Scientists and clinicians have been working to develop strategies to transiently open the BBB in a safe manner, with minimal adverse side effects. For example, intra-arterial infusion of mannitol at hyperosmotic concentrations causes massive BBB disruption. In response to hyperosmotic plasma concentrations, endothelial cells shrink and opening of the paracellular space is achieved [7,8]. Although used clinically [9], this procedure requires anesthesia, significant resources, and is limited by the frequency of procedures administered to a single patient, partly due to significant morbidity [10]. Another approach involves focused ultrasound, administered to the brain in combination with intravenous administration of encapsulated gas-filled microbubbles. In response to sonication, the bubbles cavitate, release energy [11], and open the barrier, likely due to disruption of tight junctions [12]. This approach has reached clinical trial phase [13,14]. However, the resultant BBB disruption may associate with injury to microvessels, microbleeds [15], and brain inflammation [16]. Thus, the field of controlled BBB opening for enhanced drug delivery is still experimental, and no method has shown both the efficacy and safety levels required for routine clinical use.

Increased permeability of the BBB has been documented in both animal models and human patients following abnormal synchronous neuronal depolarization, including seizures [17,18,19,20,21,22,23] and spreading depolarizations [24]. While the detailed mechanisms underlying neuronal hyperexcitability-induced BBB opening are only partly understood, recent in vitro [25,26] and in vivo [27] studies have suggested modulation of BBB permeability by the excitatory neurotransmitter, glutamate, mediated by N-methyl-D-aspartate receptors.

Transcranial magnetic stimulation (TMS), a non-invasive brain stimulation approach, generates short (50–500 μs) magnetic field pulses, inducing an electric field which depolarizes large groups of neurons when delivered at appropriate magnitude and orientation [28]. Deep TMS, a recently developed technology enabling stimulation of deeper brain regions than the standard TMS [29], has been approved for the treatment of depression [30] and obsessive compulsive disorder [31]. By stimulating neurons, TMS leads to the release of glutamate to the extracellular space [32]. In a recent study, we tested the potential of repetitive TMS (rTMS) to increase BBB permeability [27]. We demonstrated that low-frequency (1 Hz) rTMS, applied at very high amplitude, increases BBB permeability in the rat cortex. In a pilot human study, we further confirmed that repetitive deep TMS increases vascular permeability to Gd-based contrast agents in over 50% of patients with malignant glioma [27]. While single-session rTMS-induced BBB opening was demonstrated, key questions including the duration of BBB opening, permeability to different molecular size agents, efficacy, and safety with repeated stimulation are still open.

In the present study, we aimed to characterize rTMS-induced BBB opening by evaluating the time window of opening and safety. We further tested the capacity of rTMS-induced BBB opening to increase brain delivery of a high molecular weight cytostatic drug. Our findings indicate a duration of <30 min from rTMS onset, in which the BBB is open. rTMS-induced BBB opening facilitated molecular transport from the circulation into brain neuropil of both a low molecular weight fluorescent tracer and a high-molecular weight cytostatic agent. Importantly, no brain injury was found following repeated stimulation sessions.

## 2. Materials and Methods

All procedures in experimental animals were approved by the Ben-Gurion University ethics committee for animal experiments. Unless otherwise specified, all materials were purchased from Sigma-Aldrich Ltd. (Rehovot, Israel).

### 2.1. Study Design

Animals were randomly selected for treatment. In the first phase of the study, we assessed the time window of rTMS-induced BBB opening and evaluated permeability to IGF-Trap. In this set of experiments, we used repeated fluorescent angiographies together with electrocorticographic recordings in the open-window preparation. In the second set of experiments, we tested the safety of repeated sessions of rTMS. We used magnetic resonance imaging (MRI) for detection of brain edema and BBB dysfunction (BBBD). Data were analyzed using in-house developed MATLAB (MathWorks, Natick, MA, USA) algorithms, as previously reported [33,34,35]. Offline MRI analysis and histological examination were performed with the examiner blinded to group assignment.

### 2.2. Open-Window Craniotomy

Surgical procedures in male Sprague–Dawley rats (283.25 ± 48.42 g body weight, mean ± standard deviation) were performed as reported [36]. Rats were deeply anesthetized by intraperitoneal administration of ketamine (100 mg/mL, 0.08 mL/100 g) and xylazine (20 mg/mL, 0.06 mL/100 g). The tail vein was catheterized, and animals were placed in a stereotactic frame under a SteREO Lumar V12 fluorescence microscope (Zeiss Ltd., Oberkochen, Germany). Body temperature was continuously monitored and kept stable at 37 ± 0.5 °C using a feedback-controlled heating pad (Physitemp Ltd., Clifton, NJ USA). Heart rate, breath rate, and oxygen saturation levels were continuously monitored using MouseOx (STARR Life Sciences Ltd. Oakmont, PA, USA). A cranial bone section (4 mm caudal, 2 mm frontal, 5 mm lateral to bregma) was opened over the right sensory-motor cortex. The dura and arachnoid layers were removed, and the exposed cortex was continuously perfused with artificial cerebrospinal fluid (ACSF) containing (in mM): 124 NaCl, 26 NaHCO_3_, 1.25 NaH_2_PO_4_, 2 MgSO_4_, 2 CaCl_2_, 3 KCl, and 10 Glucose (pH 7.4).

### 2.3. Noncovalent Conjugation of Succinimidyl Ester (NHS) to IGF-Trap

The fluorescent tracer succinimidyl ester (NHS, 1 mg/mL in dimethyl sulfoxide, Thermo Fisher Scientific Ltd., Waltham, MA, USA) was conjugated in vitro to IGF-Trap (Trap 3.3, ~400 KDa) [37] by way of noncovalent interaction. Briefly, the protein was diluted in phosphate buffered saline (PBS) and was added with carbonate buffer (pH = 9.3) at a ratio of 1:9 (in favor of protein). A working solution was then formed by combining NHS and protein (in carbonate buffer added PBS) at a molar ratio of 1:14–1:10 (in favor of NHS). The working solution was then incubated at room temperature with slow mixing and was finally filtered with 100 kDa filter tubes (Merck Ltd., Burlington, MA, USA), by centrifuging (25 °C, 4000 rpm, 20 min), to keep only the protein-conjugated NHS. Prior to in vivo experimentation, the working solution was added with PBS to maintain required concentration.

### 2.4. Fluorescent Angiography for Assessing Vascular Permeability

Direct dynamic imaging of regional cerebral blood flow and BBB permeability measurements were performed as reported [34,36,38]. The non-BBB-permeable fluorescent tracers, sodium fluorescein (NaFlu, 376 Da), and NHS conjugated-IGF Trap (NHS-IGF-Trap) were injected i.v. (1 mg/mL in 0.9% NaCl, 0.35 mL/injection, and 20 mg/kg in PBS, respectively). Full-resolution (512 × 512 pixel) images were obtained from the exposed cortical surface (with excitation at 470 ± 35 nm, 5 frames/s for NaFlu and 1 frame/s for NHS-IGF-Trap, using CMOS camera, Andor Technology, Belfast, UK), before, during, and after injection of the tracer (Supplementary Materials Figure S1A). Offline image analysis was carried out using in-house developed MATLAB algorithms and included resizing (128 × 128 pixel), image registration [39], and segmentation using noise filtration, hole-filling, and adaptive threshold to produce a binary image, separating blood vessels from extravascular (EV) regions (Figure S1B). A primary vessel was then manually selected (Figure S1B). A BBB permeability index (PI) was calculated as follows: For NaFlu, signal intensity changes over time and space were analyzed so that each pixel was represented by an intensity vs. time (IT) curve. The mean intensity values in each compartment were used to create the compartmental IT curve (Figure S1C). The PI was defined as the ratio between EV IT curve and the primary vessel’s IT curve (vascular input function—VIF), from the initial, post-maximum point where the signal’s derivative is between 0 and −1, to the end of the measurement (tracer clearance phase, ~250–300 s):$\;PI=\frac{1}{T}\int_{t_{cr}}^{t_{end}}\frac{I_{EV}}{I_{VIF}}\left(t\right)dt\;,\;T=t_{end}-t_{cr}$. For NHS-IGF-Trap (which does not exhibit clearance from the vascular compartment), signal histograms were calculated for the primary vessel and EV compartments (Figure S1D), indicating the relative frequency by which specific signal intensity bands are found in the selected compartment. A weighted signal intensity vector was created by multiplying signal intensities with their corresponding relative frequencies—$x_{i}f_{i}$ (where $x_{i}$ is the signal intensity and $f_{i}$ the relative frequency). The weighted vectors were then ranked according to the Mann–Whitney U (MWU) ranking system [40]. The PI was defined as the ratio between the mean rank of the EV vector, and the mean rank of the primary vessel’s vector: $PI=\frac{\frac{1}{N}\sum_{1}^{N}MWU{\left(x_{i}f_{i}\right)}_{EV}}{\frac{1}{M}\sum_{1}^{M}MWU{\left(x_{i}f_{i}\right)}_{vessel}}$. The PI (Figure S1E) indicates the tracer’s transfer level from the vessel to a chosen pixel or to the EV space. To determine the impact of rTMS on brain vascular permeability, the change in PI between post- and prestimulation (baseline) was calculated. BBBD was considered when the change in PI was >3 standard deviations of the change seen following sham stimulation (see below) [27]. This method was validated in well-established models of BBB dysfunction such as cortical perfusion of sodium deoxycholate [41], photo-induced stroke [36,38], and repeated seizures [27].

### 2.5. Repetitive Transcranial Magnetic Stimulation

rTMS was applied to the anesthetized rat using a Rapid^2^ stimulator (Magstim Ltd., Oxford, UK) and a conventional circular coil (58 mm outer diameter, 56 mm inner diameter, 1.5 mm thickness, 18.4 µH inductance, Brainsway Ltd., Jerusalem, Israel) [42], placed on top of the rat’s head. One side of the coil’s outer perimeter was placed on top of the right hemisphere, perpendicular to the posteroanterior midline of the cranium, with the contralateral side elevated from the head. This orientation induced current parallel to the cortex, in the medial to lateral axis [43], and generated contralateral (and not ipsilateral) activation of the forelimb. rTMS was applied at 1 Hz (low frequency), 130% of resting motor threshold (rMT—the minimal magnetic field intensity required to elicit movement of the forelimb contralateral to the stimulated hemisphere, with no movement of the ipsilateral forelimb), with 360 µs pulse duration, 50 s train duration, 60 s inter-train interval, 5 trains, and 250 pulses in total. Total session duration was ~8.2 min. Sham rTMS was applied by diverting stimulator current from the head coil to an adjacent coil, placed 100–120 cm away. Sham stimulation did not elicit any motor response.

### 2.6. Electrocorticography Recording and Analysis

Electrocorticography (ECoG) was recorded using a telemetric system (Data Sciences International Ltd., St. Paul, MN, USA) as described [44]. A bi-polar transmitter was implanted with one electrode attached to an intracranial screw adjacent to the exposed cortex, and the second placed over the exposed cortex while secured with bone wax (Ethicon Ltd., Somerville, NJ, USA) and dental cement (GC America Ltd., Alsip, IL, USA). In-house developed MATLAB algorithms were used to display signals and for post-processing. The signal x(t) was sampled at 200 Hz and filtered using a simulated Butterworth filter, so as to display only the 1–40 Hz band. Mean spectral power (MSP) was calculated as $\bar{S}=\frac{1}{△f}\int^{\;}S\left(f\right)$, where $S\left(f\right)=\int_{-\infty }^{\infty }E\left[x\left(t\right),x\left(t+\tau \right)\right]e^{-j2\pi ft}dt$ and E[x(t)] is the signal’s expectancy. The dominant frequency (DF) was calculated as the frequency corresponding to the maximum value of S(f). Signal energy was calculated as $\int_{-\infty }^{\infty }{\left|x\left(t\right)\right|}^{2}dt$. Baseline recording was performed prior to TMS application, from which a period (12.7 ± 1.06 min, mean ± squared error of the mean) was selected as representing quiescence. Shifts in MSP, energy, and DF were then calculated in comparison to quiescence, for 1 min intervals. Statistical analysis was applied to shifts in ECoG features [45,46] as values were ranked according to the MWU ranking system [40]. Percentage difference in mean between post-TMS onset and baseline was calculated for all three features. Additionally, percentage difference in mean between non-quiescence and quiescence baseline intervals was calculated.

### 2.7. Evaluation of rTMS-Induced Brain Injury

Rats were randomly assigned to “real” or “sham” stimulation groups. Animals were anesthetized daily by intraperitoneal administration of ketamine (100 mg/mL, 0.08 mL/100 gr) and xylazine (20 mg/mL, 0.06 mL/100 gr) and were subjected to rTMS over an intact scalp (*n* = 10 for “real” and *n* = 6 for “sham”). On day 5, brain MRI (0.7T, Aspect Imaging Ltd., Shoham Israel) scans were conducted, following stimulation, under anesthesia (1–2% isoflurane with 100% O_2_). Following MRI, brains were extracted, kept in paraformaldehyde (4% in PBS) for 48 h, and then transferred to store solution (0.01% paraformaldehyde) before being sectioned for histological examination. As positive control, images were compared to those of animals subjected to photo-induced thrombotic stroke (photothrombosis) [47] via i.v. administration of 4,5,6,7-Tetrachloro-3′,6′-dihydroxy-2′,4′,5′,7′-tetraiodo-3*H*-spiro[isobenzofuran-1,9′-xanthen]-3-one (Rose Bengal, 7.5 mg/mL in 0.9% NaCl, 0.133 mL/100 gr) [36,38], while the intact cranium was exposed to halogen illumination [17]. Brain scans in photothrombosis-induced animals were performed 24 h following infarct induction (Figure S2A). Brain edema was evaluated using fast spin-echo T2-weighted imaging (TR/TE/NEX  =  3400 ms/74 ms/4) [17,20]. Offline analysis was performed following image segmentation (Figure S2B), acquired by calculating brain and peripheral signal histograms and identifying voxel intensity within brain signal range and out of peripheral signal range. Total region of hyperintense brain signal was detected (exampled by lesion detection 24 h following photothrombosis, Figure S2C,D) using in-house developed MATLAB algorithms. Briefly, the signal histogram in a 3 × 3 environment around each voxel (environmental signal) was calculated and compared to the signal histogram of a reference control region acquired from the hemisphere contralateral to the stimulated/infarct-induced hemisphere (Figure S2C). Voxels with hyperintense signal (Figure S2D) were defined as having a higher (according to MWU rank) self and mean environmental signal than the control signal (Figure S2C). All calculations were performed with in-house developed MATLAB algorithms. As hyperintense signal level measurement, the relative volume of hyperintense signal (#of hyperintense signal voxels/# of brain voxels) was calculated.

BBB permeability was evaluated using a contrast-enhanced dynamic scan protocol (DCE-MRI) as reported [33]. Seven consecutive spin echo T1-weighted scans (TR/TE/NEX  =  400 ms/14 ms/2) were performed, in which the gadolinium (Gd)-containing contrast agent, gadoteric acid (DOTAREM, Lantheus Ltd., Billerica, MA, USA), was administered i.m. (1 mL/kg) following the 1st scan (Figure S2E). Offline image registration [39], segmentation, and subtraction of post-contrast agent administration imaging from preadministration imaging were performed. A linear regression was then fitted to the data of per-voxel time-dependent magnetization, in which the slope value (Figure S2F) was considered an indication of tracer washout (slope ≤ 0) or accumulation (slope > 0) and an intact or impaired BBB, respectively [33,34,35]. Brain voxels with an abnormally high-level slope value (BBB dysfunction, BBBD, Figure S2F–G) were considered as those exhibiting statistically significant (*p* < 0.05) regression variables, positive slope and higher (according to MWU rank) self and mean environmental (3 × 3 around each voxel) slope values than the slope calculated for the temporal muscle, a non-BBB-containing tissue (Figure S2E). All calculations were performed with in-house developed MATLAB algorithms. As measurements of vascular permeability level, the relative volume of BBBD (# of BBBD voxels/# of brain voxels) was calculated. Brains of real and sham rTMS-treated animals were sectioned to 30 µm slices. Slices were placed in H_2_O for 1 min and then incubated in (9-dimethylamino-10-methyl-benzo[a]phenoxazin-5-ylidene)-ammonium chloride (cresyl-violet, 0.1% in H_2_O) for staining. Following two rinses in H_2_O to remove unbound stain, slices were differentiated by incubation in graded concentrations of EtOH: 50% for 3 min, acidified 70% for 1–2 min until slices were differentiated, 95% for 2 min, and 100% for 5 min repeated twice. Finally, slices were twice incubated in dimethylbenzene (xylene) for 5 min, and immediately mounted on slides and covered with coverslips. Slice examination was performed with light microscopy (AxioZoom, Axiocam MRc 5, Zeiss Ltd., Oberkochen, Germany).

### 2.8. Statistical Analysis

Unless otherwise specified, mean ± squared error of the mean (SEM) are reported. All comparisons were made using two-tailed Mann–Whitney-U or Wilcoxon tests (Mann–Whitney or Wilcoxon respectively, see text). *p* = 0.05 was defined as the level of significance. Statistical analysis was performed using SPSS 20 (IBM Ltd., Armonk, NY, USA).

## 3. Results

### 3.1. rTMS-Induced BBB Opening Is Transient

**Figure 1:**
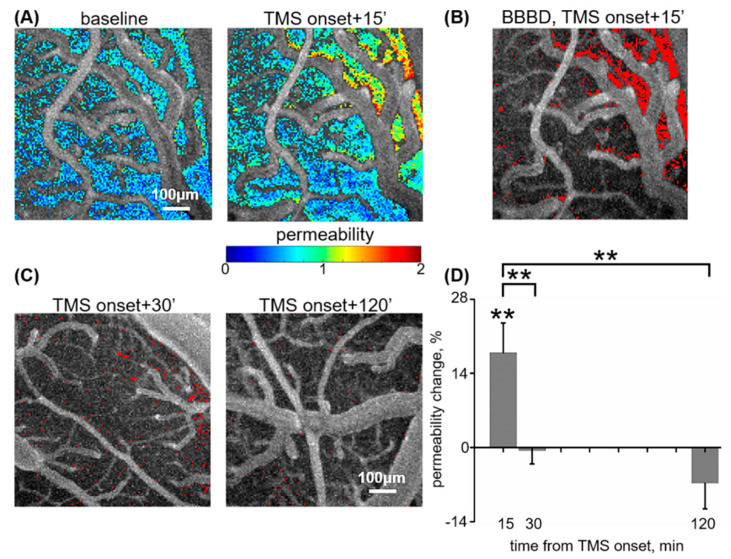
Low-frequency repetitive transcranial magnetic stimulation (rTMS)-induces blood–brain barrier (BBB) opening is time-dependent. (**A**). Fluorescent angiography of the right, exposed motor cortex of the rat, overlaid with extravascular permeability index (see Section 2), at baseline (left) and 15 min from low-frequency rTMS onset (right). (**B**)**.** Detection of BBB dysfunction (BBBD). Fluorescent angiography, at 15 min from TMS onset, overlaid with detection of extravascular pixels (red) in which permeability has changed from baseline in >3 standard deviations of the mean change seen following sham stimulation [27]. Magnification is identical to (**A**). (**C**)**.** Similar analysis as in B, performed for 30 min (left) and 120 min (right) from TMS onset. (**D**)**.** Mean ± squared error of the mean (SEM) change in permeability index relative to baseline for 15 [27], 30, and 120 min from TMS onset. ** *p* < 0.01.

The time period in which elevated vascular permeability lasts after 1 Hz rTMS at 130% of rMT (low frequency, very high amplitude) was measured using fluorescent angiography for direct imaging of pial micro-vessels. BBB permeability to the fluorescent tracer, NaFlu, was tested prior to (baseline), and at 15, 30, and 120 min following TMS onset. Permeability change from baseline was calculated for each time point. The early increase (15 min from TMS onset) in PI was 17.9 ± 5.62% compared to baseline (*n* = 13, *p* < 0.001, *p* = 0.002 compared to sham stimulation; the latter resulted in 0.8 ± 1.86% PI shift, *n* = 14, *p* = 0.49 compared to baseline, Mann–Whitney, Figure 1A,B,D) [27]. At 30 min from TMS onset, PI shift decreased (*p* = 0.008, Mann–Whitney) to −0.62 ± 2.48% (*n* = 7, *p* = 0.63 compared to baseline, *p* = 0.77 compared to sham, Mann–Whitney, Figure 1C,D). At 120 min from TMS onset, PI shift dropped (*p* = 0.005, Mann–Whitney) to −6.76 ± 4.85% (*n* = 7, *p* = 0.15 compared to baseline, *p* = 0.14 compared to sham, Mann–Whitney, Figure 1C,D). These findings indicate that low-frequency rTMS induces BBB opening within minutes from the onset of stimulation and that normalization of vascular permeability occurs at 15–30 min from TMS onset (or ca. 7–22 min from TMS offset).

### 3.2. Reversible ECoG Depression Coincides with BBB Normalization

**Figure 2:**
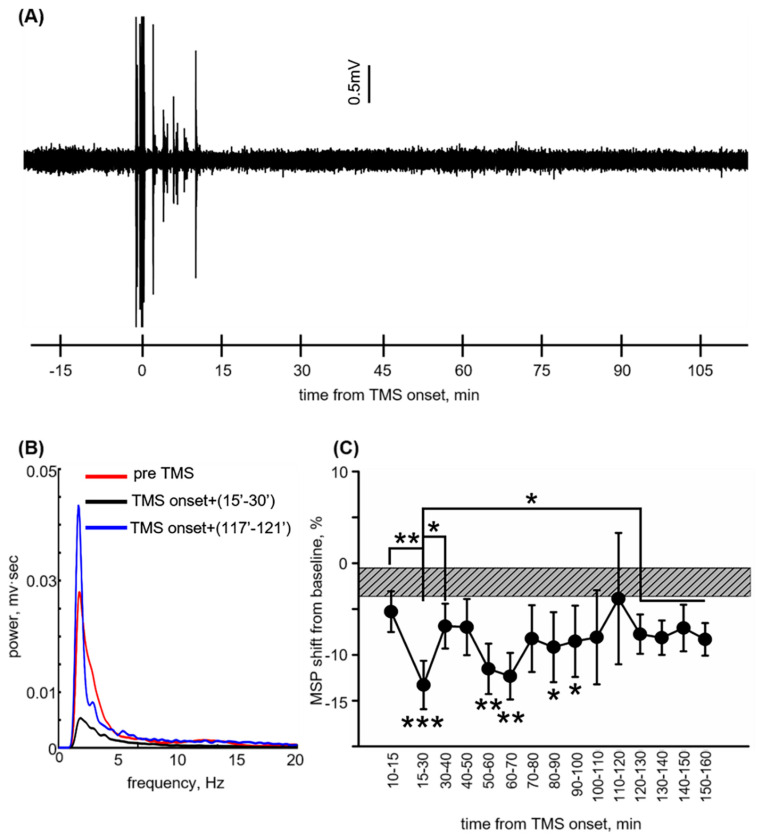
Electrocorticography (ECoG) recording indicates reversible post-stimulation depression. (**A**) Electrocorticography of an anesthetized rat during rTMS; 0 min = rTMS onset. (**B**) Mean signal spectrum at pre rTMS (baseline, red),15–30 min (black), and 117–121 min (blue) post-TMS onset. (**C**) Mean ± SEM spectral power (MSP) shift from baseline at different time points after TMS onset. Grey box indicates mean ± SEM baseline spectral power shift. * *p* < 0.05, ** *p* < 0.01, *** *p* < 0.001.

ECoG recordings (Figure 2A) from rats subjected to rTMS at 1 Hz, 130% of rMT (*n* = 22) were analyzed to evaluate an impact of low-frequency stimulation on cortical electrical activity (see Section 2). The duration of the stimulation session was ~8.2 min. At 15–30 min from TMS onset, energy and mean spectral power dropped compared to baseline (−15.58 ± 3.04%, and −13.3 ± 2.65%, *p* < 0.001 and *p* = 0.003, respectively, and *p* = 0.009 compared to 10–15 min from TMS onset, Mann–Whitney Figure 2B,C). The decrease from baseline continued until 100 min from TMS onset, followed by an incline at 120–130 min (−7.81 ± 2.11% and −7.74 ± 2.15%, *p* = 0.02 and *p* = 0.03 in comparison to 15–30 min from TMS onset, *p =* 0.13 and *p* = 0.16 in comparison to baseline, Figure 2C), which remained stable until 160 min from TMS onset. No change from baseline was found for dominant frequency at all time intervals (data not shown). These findings indicate reversible ECoG depression, which coincides with the return to normal permeability.

### 3.3. Low-Frequency rTMS Increases Cortical Vascular Permeability to IGF-Trap

To test for potential therapeutic implications of rTMS-induced BBB opening, we tested the effect of stimulation on the extravasation of IGF-Trap—an IGF-targeting protein designed to block tumor progression [6].

**Figure 3:**
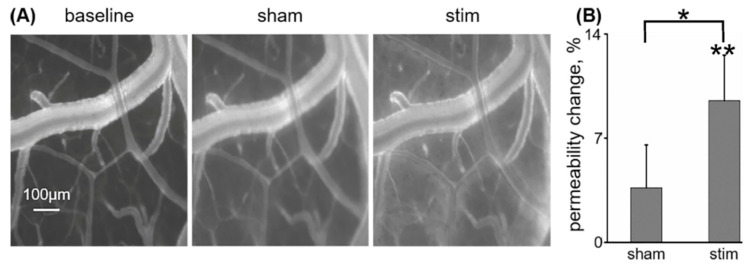
Low-frequency rTMS increases neocortical vascular permeability to IGF-Trap: (**A**) Fluorescent angiography of the exposed anesthetized rat cortex, following intravenous administration of succinimidyl ester-conjugated IGF-Trap (20 mg/kg), at prestimulation (baseline, left), following sham rTMS (sham, center) and following real rTMS (stim, right) at 1 Hz, 130% of resting motor threshold. Extravasation of fluorescent IGF-Trap into extravascular space, following real stimulation, is apparent. (**B**) Mean ± SEM increase in permeability index (see Section 2) from baseline, for post-sham and post-real stimulation. * *p* < 0.05, ** *p* < 0.01.

For assessment of BBB permeability, NHS (see Section 2)-labelled IGF-Trap was intravenously injected (*n* = 5). Imaging was performed at baseline, 15 min from the onset of sham rTMS (1 Hz, delivered at 35% of stimulator output), and 15 min from the onset of real rTMS (1 Hz, 130% of rMT). While sham rTMS did not elicit a change in cortical vascular permeability to fluorescently labeled IGF-Trap (3.67 ± 2.88%, *p* = 0.58 in comparison to baseline, Mann–Whitney, Figure 3A,B), real rTMS resulted in its leakage and accumulation in the perivascular space (9.52 ± 3.06%, *p* = 0.005 compared to baseline, Mann–Whitney, *p* = 0.04 compared to sham rTMS, Wilcoxon, Figure 3A,B).

### 3.4. Repeated Low-Frequency rTMS Does Not Induce Brain Injury

MRI was performed following 5 days of real (*n* = 10) or sham (*n* = 6) 1 Hz rTMS delivered at 130% of rMT. Two of the ten real-stimulated animals died during anesthesia prior to treatment completion (days 2 and 5), while a single sham-stimulated animal died (day 4) (*p* = 0.68, Mann–Whitney). All rats lost weight during the five days of repeated treatment under general anesthesia. No difference in weight loss was observed between real and sham groups (−3.8 ± 1.43% for real, −6.98 ± 1.5% for sham, *p* = 0.07, Mann–Whitney).

**Figure 4:**
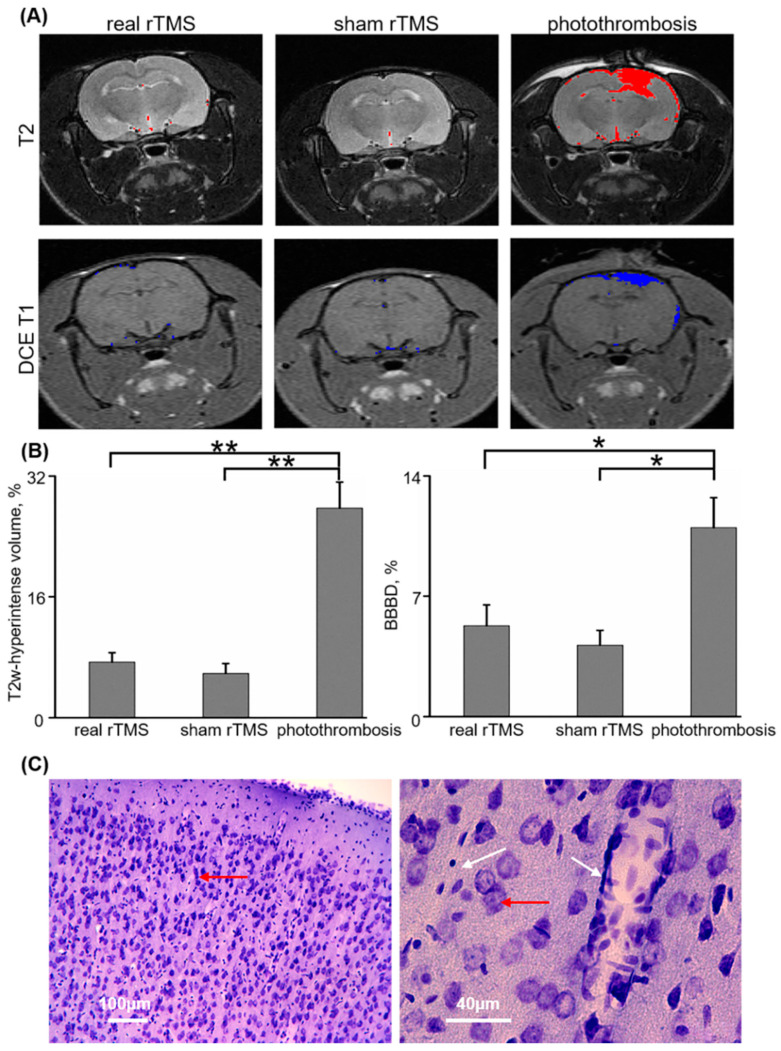
Repeated low-frequency rTMS does not result in brain injury: (**A**) Coronal T2-weighted (top) and T1-weighted (bottom) MRI of the rat head following 5 consecutive days of low-frequency rTMS, one session/day, delivered as either real (left) or sham (center) stimulation. Imaging was also carried out in animals that underwent photothrombosis-induced stroke, at 24 h post-infarct induction (right). Offline detection of hyperintense voxels in T2-weighted imaging (red, top, see Section 2) and BBB dysfunction voxels in T1-weighted imaging (blue, bottom) are overlaid. (**B**) Left: mean ± SEM relative volume of hyperintense brain region in T2-weighted (T2w) imaging. Right: mean ± SEM relative BBB dysfunction (BBBD) volume in dynamic contrast-enhanced (DCE) T1-weighted imaging. (**C**) Microscopic images (left: ×10, right: ×40) of coronal slices dissected from the right primary motor cortex in real rTMS-treated animals, stained with cresyl-violet (see Section 2). Neuronal cell bodies (red arrow) appear alongside brain vasculature (white arrow). * *p* < 0.05, ** *p* < 0.01.

Brain volume with hyperintense signal was measured using T2-weighted imaging. No differences in relative volume of hyperintense brain signal were found between real and sham groups (7.34 ± 1.25%, *n* = 8, vs. 5.84 ± 1.31%, *n* = 5, respectively, *p* = 0.46, Mann–Whitney, Figure 4A,B). In contrast, analysis of brains exposed to a photothrombotic stroke resulted in higher relative hyperintense volume compared with either real or sham stimulation groups (27.74 ± 3.45%, *n* = 7, *p* = 0.001 and *p* = 0.003 in comparison to real and sham, respectively, Figure 4A,B).

DCE-MRI was applied for the evaluation of long-lasting microvascular injury and a leaky BBB. No difference in brain volume with BBB dysfunction was found between real and sham groups (5.28 ± 1.21% vs. 4.14 ± 0.87%, *p* = 0.66, Mann–Whitney). This is in contrast to scans from animals after photothrombotic stroke, which was associated with a significantly higher brain volume with BBB dysfunction, compared to that found for both real and sham stimulation groups (10.99 ± 1.74%, *p* = 0.02 in comparison to both real and sham, Figure 4A,B).

Histological examination of brains subjected to repeated rTMS, following staining with cresyl-violet, confirmed that the stimulated cortex appeared to be of normal structure with no ischemic damage or microhemorrhages (Figure 4C).

## 4. Discussion

We report that rTMS-induced BBB opening is transient, safe, and effective in facilitating delivery of an anticancer pharmaceutical. Vascular permeability to NaFlu was significantly higher for <30 min from TMS onset. Parallel ECoG recordings indicate reversible, short-spanned depression of cortical activity. We further demonstrate that rTMS results in increased vascular permeability to the cytostatic agent, IGF-Trap. Using MRI analysis and histological examination, we report that 5 days of repeated stimulation do not lead to an apparent injury to brain tissue or long-lasting increase in vascular abnormality.

We first measured the time window from TMS onset, in which the BBB was open, as it would be the relevant time window for drug administration. Our results indicate a rapid opening and closing of the barrier following stimulation, with an effective time-window of <30 min following TMS onset. These results suggest that in order for rTMS-induced BBB opening to be effective for brain drug delivery, drug administration must be coupled to the stimulation session and should be applied in a manner that will produce maximal serum concentrations within that time frame. These results are similar to ones previously reported for focused ultrasound, which induces a rapid BBB opening that lasts for <40 min [48]. In contrast, hyperosmotic opening of the barrier with mannitol was shown to last for up to 6 h [49].

We also applied here telemetric ECoG recordings to test for alterations in cortical activity following rTMS. Recording was limited to baseline (prestimulation) and following stimulation, due to the noise induced by the magnetic field. Immediately after stimulation, however, a clear suppression of activity was found, peaking at 15–30 min from TMS onset and lasting >100 min. Notably, peak suppression of brain activity was associated with the reduction in vascular permeability, around 15–30 min from TMS onset. Inhibition of neuronal activity by low-frequency rTMS was demonstrated earlier [50,51] and was suggested to be mediated by gamma amino butyric acid B (GABA-B) receptors [52,53]. However, a reverse effect of low-frequency stimulation was also reported [54], suggesting that the inhibition is not protocol-specific. To what extent the suppression in activity is due to post-tetanic depression or directly related to shifts in permeability is not known. Either way, future studies are required to verify to what extent measuring surface brain activity could serve as a clinically feasible approach to follow BBB opening and help in timing the administration of therapeutics. This is especially important as rTMS may not affect all patients to the same extent [27].

We report, for the first time, that rTMS-induced BBB opening is sufficient to facilitate the extravasation of the cytostatic protein, IGF-Trap, from the circulation. IGF-Trap is a potent inhibitor of several highly aggressive carcinoma cell types [5,6]. The IGF axis consists of the IGF-I receptor and its high-affinity binding ligands IGF-I and IGF-II. These agents have been implicated in all stages of cancer growth and progression [37,55,56,57,58], as well as in the regulation of tumor microenvironment, and identified as prognostic markers and therapeutic targets in brain tumors such as glioblastoma multiforme. Our findings indicate the ability of rTMS to generate BBB opening to an anticancer pharmaceutical and imply that anticancer treatment can be coupled to TMS to achieve increased efficacy. Future animal studies should confirm that cytostatic therapy during TMS slows the progression of brain metastases or primary malignant tumors.

Finally, we characterized the safety level of repeated, low-frequency rTMS sessions. Five days of repeated rTMS did not result in a change in animal behavior or weight gain/loss compared to sham controls. MRI and histological examination confirmed an apparent normal healthy brain. In contrast to mannitol or focused ultrasound-induced BBB opening, no marked edema [59], microbleeds [15] or damage to brain structures were observed. Together, the lack of brain vascular abnormalities found here, alongside reversible, short-spanned BBB opening with a transient depression of neuronal activity, suggest that modulation of BBB opening by low-frequency rTMS is not accompanied by long-standing brain abnormalities. Future toxicological and functional studies, in larger mammals, are awaited to confirm the safety of repeated, high-intensity rTMS for drug delivery.

## 5. Conclusions

We demonstrate the translational potential and feasibility of rTMS-induced BBB opening. Awareness of TMS has rapidly grown, especially with regard to its ability to treat neuropsychiatric diseases such as depression [30] by manipulating regional neuronal excitability and neural plasticity [60]. However, applying the technique to manipulate BBB permeability is novel. Future preclinical studies are awaited to evaluate additional safety and efficacy [61] and test vascular permeability to additional chemotherapeutics and optimization of stimulation parameters to maximize barrier opening without compromising safety. Future clinical studies should evaluate the therapeutic efficacy of this approach and patient outcome.

## Data Availability

The datasets used and/or analyzed during the current study are available from the corresponding author on reasonable request.

## Supplementary Materials

The following are available online at https://www.mdpi.com/1999-4923/12/10/946/s1, Figure S1: Dynamic fluorescence imaging for BBB permeability quantification: (A) Fluorescence imaging of an exposed rat cortical section following intravenous administration of sodium fluorescein (see Section 2). (B) Image segmentation indicating extravascular regions (blue). A primary vessel is manually selected (red and white frame). (C) Intensity vs. time curves, calculated for the primary vessel (red) and the extravascular space (blue). (D) Signal histograms calculated for the primary vessel (red) and extravascular space (blue). (E) Per-pixel, sodium fluorescein permeability index for the extravascular space. Figure S2: T2 and DCE T1-weighted MRI for detection of brain injury and BBB dysfunction: (A) T2-weighted coronal MRI of the rat head, 24 h following photo-induced thrombotic stroke (see Section 2). (B) Image segmentation excluding brain region (blue). (C) Manual selection of a reference control region (green). (D) Detection of hyper-intensified voxels (red) in comparison to control (see Section 2). (E) T1-weighted coronal MRI of the rat head, 24 h following photo-induced thrombotic stroke, without contrast enhancement (pre-Gd, left) and following 6 consecutive scans post-systemic gadoteric acid administration (post-Gd, right). The temporal muscle (green) is selected for calculation of reference vascular permeability of a non-BBB-containing tissue (see Section 2). (F) Per-voxel magnetization vs. time is fitted with a linear regression in which the slope (color-coded to scale bar; BBB dysfunction—BBBD values are presented) is a direct indication of BBB permeability. (G) Voxels with abnormally high permeability levels (BBBD, see Section 2) are detected (blue).

## Abbreviations

ACSF:	Artificial cerebrospinal fluid
BBB:	Blood–brain barrier
BBBD:	BBB dysfunction
CNS:	Central nervous system
DCE:	Dynamic contrast-enhanced
DF:	Dominant frequency
ECoG:	Electrocorticography
EV:	Extravascular
IGF:	Insulin-like growth factor
IT:	Intensity vs. time
MRI:	Magnetic resonance imaging
MSP:	Mean spectral power
MWU:	Mann–Whitney U
NaFlu:	Sodium fluorescein
NHS:	Succinimidyl ester
PBS:	Phosphate buffered saline
PI:	Permeability index
RB:	Rose Bengal
rMT:	Resting motor threshold
rTMS:	Repetitive transcranial magnetic stimulation
SEM:	Squared error of the mean
