# Supplementary figures and images for: TMS-Induced Controlled BBB Opening: Preclinical Characterization and Implications for Treatment of Brain Cancer
Source: Pharmaceutics. 2020 Oct 5;12(10):946. doi: 10.3390/pharmaceutics12100946 (PMC7650663; doi:10.3390/pharmaceutics12100946)

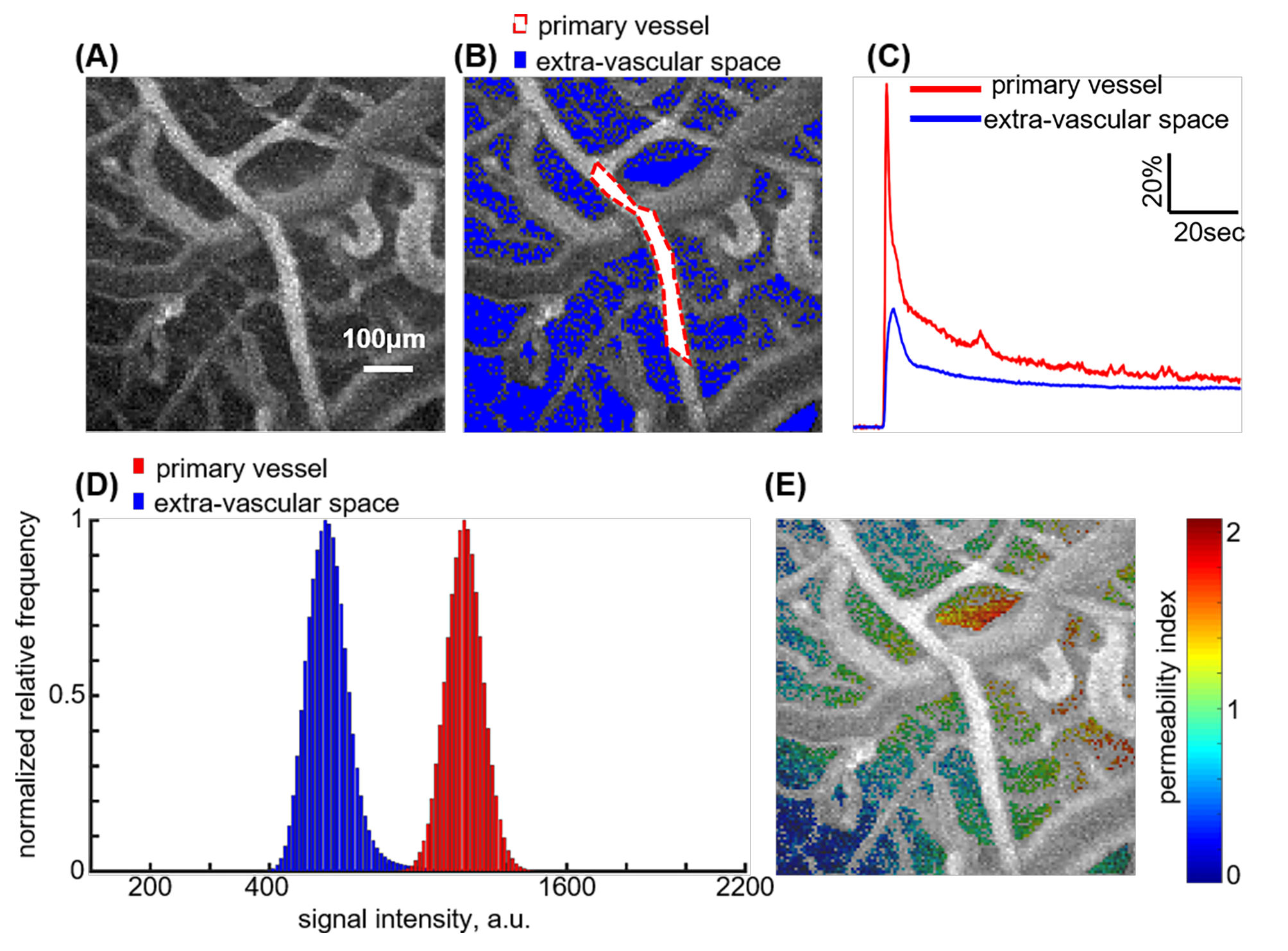

Supplement: Supplementary file 1 [file pharmaceutics-12-00946-s001.zip › Figure S1.tif]

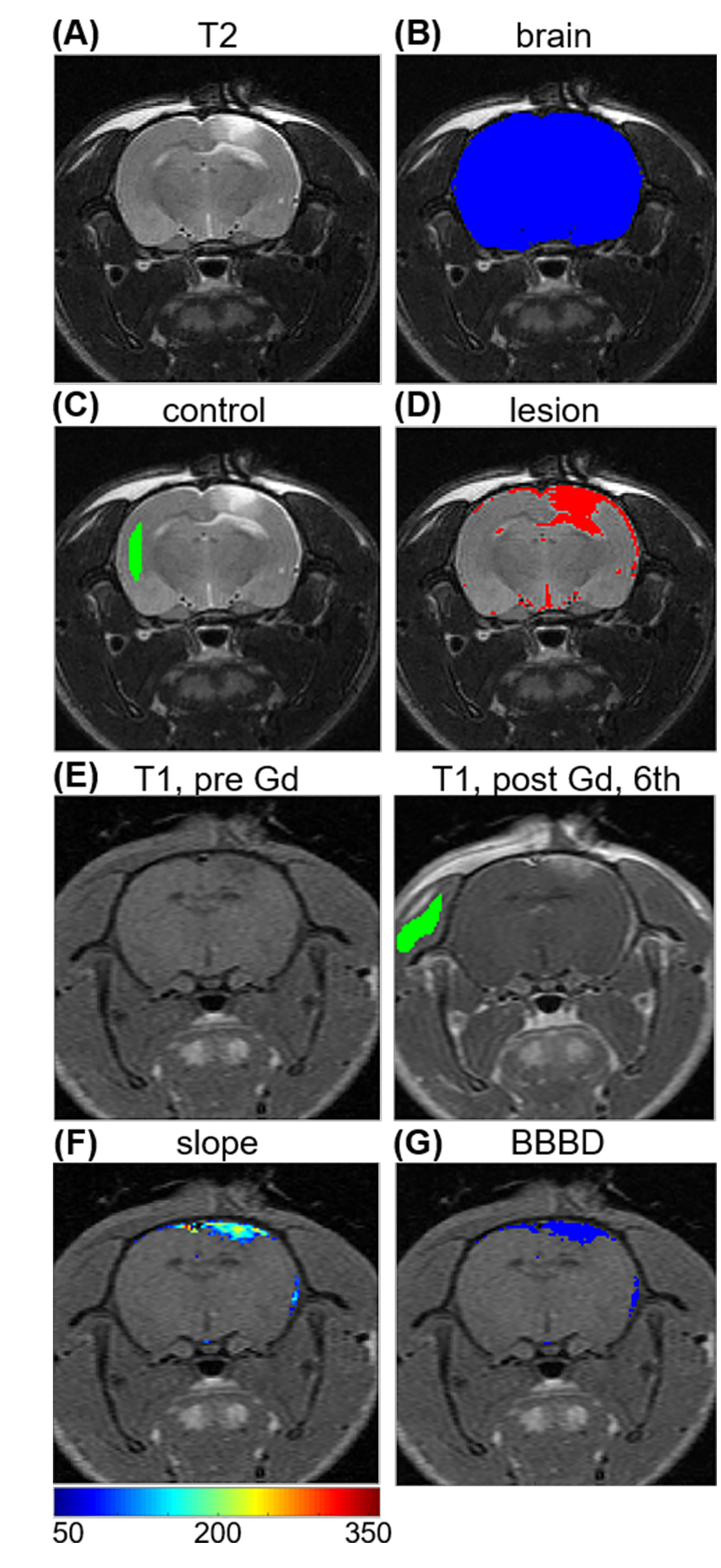

Supplement: Supplementary file 1 [file pharmaceutics-12-00946-s001.zip › Figure S2.tif]
